# Myeloid-derived suppressor cell depletion therapy targets IL-17A-expressing mammary carcinomas

**DOI:** 10.1038/s41598-020-70231-7

**Published:** 2020-08-07

**Authors:** Bassel Dawod, Jinghua Liu, Simon Gebremeskel, Chi Yan, Antonia Sappong, Brent Johnston, David W. Hoskin, Jean S. Marshall, Jun Wang

**Affiliations:** 1 Canadian Center for Vaccinology, Dalhousie University, Halifax, NS Canada; 2 Department of Microbiology and Immunology, Dalhousie University, Halifax, NS Canada; 3 Department of Pediatrics, Dalhousie University, Halifax, NS Canada; 4 Department of Pathology, Faculty of Medicine, Dalhousie University, Halifax, NS Canada; 5 Canadian Center for Vaccinology, IWK Health Centre, Research and Clinical Care Pavilion, 3rd Floor West, 5850/5980 University Avenue, Halifax, NS B3K 6R8 Canada; 6 Beatrice Hunter Cancer Research Institute, Halifax, NS Canada

**Keywords:** Breast cancer, Cancer microenvironment, Cancer models, Interleukins, Immune evasion

## Abstract

Triple-negative breast cancer (TNBC) is an invasive subtype of breast cancer but paradoxically associated with increased tumor-infiltrating leukocytes. The molecular and cellular mechanisms underlying TNBC immunobiology are incompletely understood. Interleukin (IL)-17A is a pro-inflammatory cytokine that has both pro- and anti-tumor effects and found in 40–80% of TNBC samples. We report here that IL-17A mRNA and protein are detectable in some human TNBC cell lines and further upregulated by IL-23 and LPS stimulation. Furthermore, the impact of tumor-derived IL-17A in host immune response and tumor growth was examined using murine TNBC 4T1 mammary carcinoma cells transduced with an adenoviral vector expressing IL-17A (AdIL-17A) or control vector (Addl). Compared to Addl-transduction, AdIL-17A-transduction enhanced 4T1 tumor growth and lung metastasis in vivo, which was associated with a marked expansion of myeloid-derived suppressor cells (MDSCs). However, AdIL-17A-transduction also induced strong organ-specific and time-dependent immune activation indicated by dynamic changes of NK cells, B cells, CD4, and CD8 T cells in peripheral blood, lung, and tumor site, as well as the plasma levels of IFNγ. Such findings highlight that tumor-associated IL-17A induces concurrent immune activation and immune suppression. Administration of anti-Gr1 or anti-G-CSF antibody effectively depleted MDSCs in vivo, markedly reducing the growth of AdIL-17A-transduced 4T1 tumors, and eliminating lung metastasis. Collectively, our study demonstrates that MDSC depletion is an effective and practical approach for treating IL-17A-enriched mammary carcinomas.

## Introduction

Interleukin (IL)-17A is the founding member of the IL-17 cytokine family containing six family members (IL-17A to IL-17F) and five receptors (IL-17RA to IL-17RE)^1,2^. Both IL-17A and its closest family member IL-17F signal through a multimeric receptor complex, consisting of IL-17RA and IL-17RC subunits, to activate downstream signalling cascades^1,2^. This leads to the production of additional pro-inflammatory cytokines (e.g., IL-6 and TNFα), chemokines (e.g., CXCL1 and CXCL2), growth factors [e.g., granulocyte-colony stimulating factor (G-CSF) and granulocyte–macrophage colony-stimulating factor (GM-CSF)], and other proteins such as matrix metalloproteinases^1,2^. IL-17A and IL-17F are mainly produced by innate immune cell subsets and/or activated type 17 T helper (Th17) cells depending on the context of the immune response^3,4^. It has been shown that the tumor microenvironment is enriched with various soluble mediators, including IL-6, TGFβ, IL-1β, and IL-23, which collectively promote Th17 differentiation via induction of master transcription factor RORγ^5,6^. Both Th17 and other IL-17A-producing cells, including IL-17A-producing cancer cells, have been detected in a variety of human and murine tumors^7–11^. However, the role of IL-17/Th17 in cancer is complex^12,13^. IL-17A has been reported to have both pro-tumor^10,14–20^ and anti-tumor effects^21–23^ in murine tumor models. Similarly, some clinical studies have found that an enhanced IL-17/Th17 immune profile is associated with increased tumor invasiveness^24–27^, while others have correlated IL-17/Th17 responses with enhanced patient survival^28,29^. Many factors, such as the tumor type, the tumor stage, and the cellular source and kinetics of IL-17/Th17 responses, may contribute to the controversy in a context-dependent manner^6,30–32^. Notably, IL-17A and IL-17F have also been shown to be constitutively expressed by mucosal tissues and markedly up-regulated upon stimulation by acute and/or chronic insults^33–35^. However, it remains unclear how tumor-derived IL-17A may shape tumor microenvironment, control anti-tumor immunity, and influence the course of tumor development and tumor progression.

Triple-negative breast cancer (TNBC) is defined by a lack of estrogen receptor (ER), progesterone receptor (PR), and human epidermal growth factors receptor 2 (HER2). It is a subtype of breast cancer that lacks targeted therapy and exhibits poor overall survival, increased chance of metastasis, and a higher likelihood of recurrence^36,37^. While clinical studies have demonstrated the prognostic and predictive importance of tumor-infiltrating leukocytes (TILs) in most subtypes of breast cancer^38–40^, TNBC patients are paradoxically found to have more tumors with intermediate or high density of TILs than non-TNBC patients in multiple clinical studies^40^. Increased density of TILs is associated with better response to neoadjuvant chemotherapy and a significantly lower risk of recurrence or death, distant recurrence, and overall mortality in TNBC patients^38–40^. Notably, not only does the amount of lymphocytic infiltration but also the phenotype of the infiltrates determine clinical outcome. Among all subtypes of breast cancer, TNBC tumors are found to be highly enriched with IL-17A^+^ cells^24,25,29^ and a Th17-related metagene profile^29^. While the IL-17A-producing cells are commonly identified as tumor-infiltrating CD4^+^ and CD8^+^ T cells^25,29^, tumor-infiltrating γδ T cells^25,41^, and tissue macrophages^42^, it is unclear if IL-17A may also be produced by breast tumor cell themselves. Given a known function of IL-17A in promoting cellular immune responses, we hypothesized that the intense IL-17A^+^ cellular infiltration found in TNBC patients might be triggered and/or attributed by tumor-derived IL-17A production. In this study, we examined the hypothesis and found that some TNBC cell lines constitutively express IL-17A at both mRNA and protein levels, which were further induced by inflammatory stimuli. Using a murine 4T1 TNBC model, we further demonstrated that ectopic IL-17A expression by tumor cells enhanced 4T1 tumor growth and metastasis. The enhanced disease severity in IL-17A-expressing tumors correlated with the marked expansion of myeloid-derived suppressor cells (MDSCs) despite the concurrent activation of NK cells, B cells, CD4, and CD8 T cells. The depletion of MDSCs with an anti-Gr1 or anti-G-CSF antibody potently attenuated the growth and metastasis of IL-17A-expressing 4T1 tumors, while the treatment only exhibited marginal therapeutic effects in preventing growth and metastasis of control 4T1 tumors. Collectively, our data highlight the pleiotropic effects of IL-17A in breast cancer and support the use of therapeutic modalities that deplete MDSCs in IL-17A-enriched tumors.

## Results

### IL-17A is constitutively expressed by some human TNBC cells and further upregulated upon IL-23 and LPS stimulation

**Figure 1:**
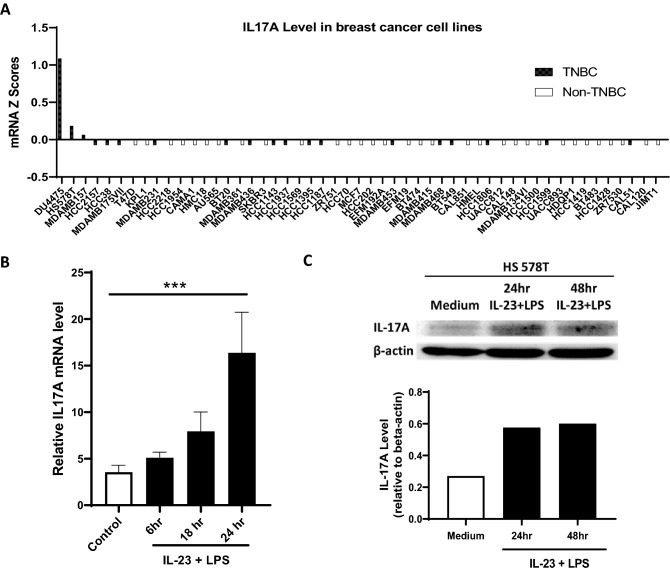
IL-17A is constitutively expressed by some human TNBC cell lines and further upregulated upon IL-23 and LPS stimulation. (**A**) IL-17A mRNA expression levels in 53 different human breast cancer cell lines, including 19 TNBC cell lines, were extracted from C-bioportal. (**B**/**C**) Human triple-negative breast cancer cell line HS578T was cultured in medium alone or stimulated with human IL-23 (10 ng/mL) plus LPS (1 µg/mL) for various times points. Total RNAs were extracted at 6, 18, and 24 h post-treatment, and IL-17A mRNA expression was examined by RT-PCR. The data is expressed as the mean ± SEM of 6–8 replicates per time point. The statistical analysis was conducted using one-way ANOVA with Dunnett’s multiple comparisons test. ***P < 0.001 comparing to untreated control (**B**). Whole-cell extracts were collected at 24 and 48 h post-treatment, and IL-17A protein was detected by western blot where β-actin was used as a loading control. The relative levels of IL-17A were determined using scanning densitometry (**C**).

To investigate whether breast cancer cells can produce IL-17A, we first queried the Cancer Cell Line Encyclopedia (cbioportal.org) that contains comprehensive molecular and genetic characterization of over 1700 human cancer cell lines, including 20 TNBC cell lines and 36 non-TNBC cell lines^43^. Remarkably, three TNBC cell lines, which account for ~ 15% of TNBC cells in the database, show detectable IL-17A mRNA levels. In sharp contrast, none of the 36 non-TNBC cell lines has detectable IL-17A (Fig. 1A), suggesting that IL-17A production may represent a unique intrinsic feature of TNBC cells. Among three IL-17A-producing TNBC cell lines (DU4475, HS578T and MDAMB157), HS578T has an intermediate level of IL-17A and was selected as a representative of IL-17A-expressing TNBC cell line for additional experiments. HS578T cells were cultured under standard culture condition or stimulated with human IL-23 and LPS for various time points. The IL-17A expression at both mRNA and protein levels were examined by RT-PCR and western blot. In strong agreement with the public database, low levels of IL-17A mRNA and protein were detected in HS578T cells under the standard culture condition. Importantly, both IL-17A mRNA and protein were significantly upregulated upon IL-23 and LPS stimulation (Fig. 1B,C). Thus, TNBC cells, at least some of them, are fully able to produce pro-inflammatory cytokine IL-17A, which may play a key role in creating a unique tumor microenvironment in TNBC patietns.

### AdIL-17A transduction promotes 4T1 tumor growth and metastasis in vivo without influencing tumor growth in vitro

**Figure 2:**
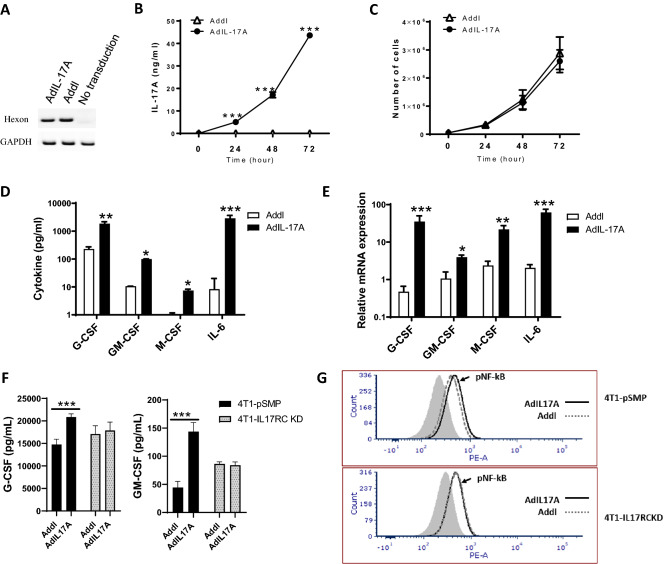
In vitro characterization of 4T1 cells upon Ad transduction. 4T1 cells were transduced with AdIL17A or Addl at MOI = 200. (**A**) Genomic DNA isolated from Ad-transduced 4T1 cells and untransduced 4T1 cells were used as PCR templates for detecting hexon and GAPDH genes. (**B**) IL17A production in Ad-transduced 4T1 cell cultures was measured by ELISA. (**C**) In vitro growth curves of Ad-transduced 4T1 cells were determined by cell counting. (**D**) The levels of G-CSF, GM-CSF, M-CSF, and IL-6 in 48-h Ad-transduced 4T1 cultures were determined by multiplex Luminex assay. (**E**) The expression of G-CSF, GM-CSF, M-CSF, and IL-6 mRNAs in Ad-transduced 4T1 cells were measured by RT-PCR and expressed relative to GAPDH. The data is presented as the mean ± SEM of quadruplicate samples from a representative experiment. *P < 0.05; **P < 0.01; ***P < 0.001 compared to Addl using the unpaired Student *t*-test. (**F**) The levels of G-CSF and GM-CSF were measured by ELISA in 4T1-pSMP and 4T1-IL-17RCKD culture supernatants at 24 h following Ad-transduction. *P < 0.05; ***P < 0.001 compared to corresponding Addl group. (**G**) The levels of phosphorylated NF-kB and STAT3 in 4T1-pSMP and 4T1-IL-17RCKD were determined at 48 h after AdIL-17A- or Addl-transduction using flow cytometry.

To examine the impact of tumor-derived IL-17A in tumor microenvironment, tumor growth, and anti-tumor immunity development, a replication-deficient adenovirus expressing an IL-17A transgene (AdIL-17A) or an empty viral vector (Addl) were used to transduce murine 4T1 TNBC cells. The two viral vectors showed comparable transduction efficiencies as indicated by levels of adenoviral hexon gene DNA in 4T1 cells transduced with AdIL-17A and Addl (Fig. 2A). The IL-17A transgene expression by 4T1 cells was efficient; more than 40 ng/mL of IL-17A was detected in culture supernatants of AdIL-17A-transduced, but not Addl-transduced 4T1 cells after 48 h in culture (Fig. 2B). Despite the expression of IL-17RA and IL-17RC on 4T1 TNBC tumor cells (Supplementary Fig. 1), 4T1 cells transduced with AdIL-17A and Addl showed similar growth kinetics in vitro (Fig. 2C), suggesting that IL-17A stimulation has a little direct effect on the growth of 4T1 TNBC cells. Compared to the cells transduced with Addl, AdIL-17A-transduced 4T1 cells had extra production of the cytokines, including G-CSF, GM-CSF, macrophage-colony stimulating factor (M-CSF) and IL-6 at 48 h post-transduction (Fig. 2D, E). In order to understand whether enhanced cytokine production by AdIL-17A-transduced cells was due to IL-17A production or bystandard effects, we also carried out the viral transductions using a stable 4T1 cell line with IL-17RC knockdown (4T1-IL-17RCKD), and a corresponding control cell line (4T1-pSMP) with intact IL-17RC generated previously^44^. We found that the increased cytokine/growth factor production (such as G-CSF and GM-CSF) and NF-κB activation in 4T1-pSMP cells upon AdIL-17A-transduction relative to Addl-transduction disappeared in 4T1-IL-17RCKD cells (Fig. 2F, G). These results explicitly demonstrate that an autocrine loop of IL-17A/IL-17R and IL-17A-induced NF-κB pathway is involved in increased cytokine/growth factor production.

**Figure 3:**
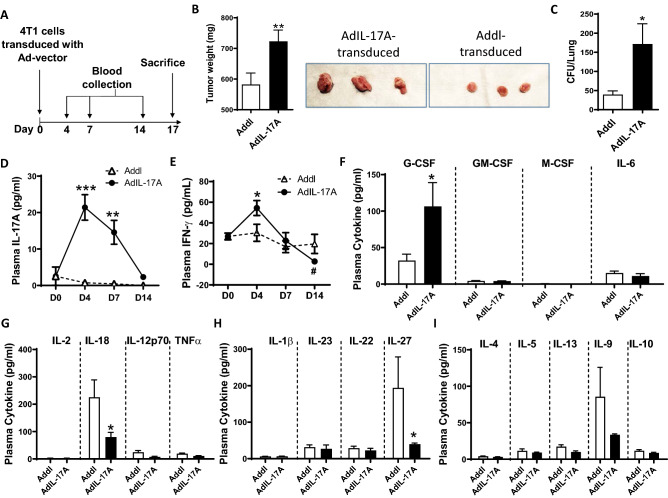
IL-17A promotes 4T1 tumor growth and lung metastasis. (**A**) Schematic diagram of the Ad-transduced 4T1 model. Mice were inoculated with 1 × 10^6^ Ad-transduced 4T1 cells; serum samples were collected on days 4, 7, and 14 post tumor injection, and mice were sacrificed on day 17. (**B**) Tumor weights were measured at day 17 (n = 25 per group, representative tumors from two groups are shown). (**C**) Lung metastases were measured by clonogenic plating assay (n = 20 per group). (**D**/**E**) Plasma levels of IL-17A and IFNγ (days 0, 4, 7, and 14) were measured by ELISA (n = 5 per group). (**F/G/H/I**) Plasma cytokine profiles (day 7) measured by multiplex cytokine array (n = 5 per group). *P < 0.05; **P < 0.01; ***P < 0.001 compared to Addl, #*P* < 0.05; Day 14 compared to Day 4 within AdIL-17A group, using two-way ANOVA with Dunnett’s multiple comparisons test (panel **D**/**E**) or unpaired Student’s *t*-test (panel **B**/**C**/**F**/**H**/**I**).

To assess how IL-17A-induced cytokine responses may influence tumor progression, we inoculated Ad-transduced 4T1 tumor cells into syngeneic BALB/c mice via orthotopic injection into the mammary fat pad (Fig. 3A). Consistent with a pro-tumorigenic role of IL-17A, mice inoculated with AdIL-17A-transduced 4T1 cells had significantly larger tumors (Fig. 3B) and elevated lung metastasis (Fig. 3C) at the sacrifice on day 17. Associated with these observations, IL-17A was transiently detected in plasma samples collected from the mice receiving AdIL-17A-transduced, but not Addl-transduced 4T1 cells (Fig. 3D). In addition, plasma IFN-γ levels exhibited dynamic changes in the mice receiving AdIL-17A-transduced 4T1 cells. Compared to the Addl-transduced counterparts, IFN-γ was increased at day 4 and subsequently suppressed at day 14 post tumor inoculation (Fig. 3E). A multiplex cytokine array was used to examine the levels of additional cytokines in plasma samples collected at day 7 post tumor inoculation (Fig. 3F–I). Plasma G-CSF levels were significantly elevated in mice receiving AdIL-17A-transduced 4T1 cells compared to the Addl counterparts (Fig. 3F). In contrast, the plasma levels of GM-CSF, M-CSF, and IL-6 were very low in both groups. Among other cytokines examined in the array, the plasma levels of IL-18 and IL-27 were significantly reduced in mice receiving AdIL-17A-transduced 4T1 cells compared to the Addl counterparts (Fig. 3G, H). Given an established role for IL-18 and IL-27 in stimulating optimal IFNγ production and Th1 responses^45,46^, our data suggest that increased tumor burden in the mice receiving AdIL-17A-transduced 4T1 cells could be due to the progressive loss of Th1 immunity.

Together, our results demonstrate that an IL-17A-expressing TNBC tumor microenvironment stimulates robust cytokine responses, particularly G-CSF production, in tumor-bearing hosts and promotes primary tumor growth and metastasis that are associated with the features consistent with immune suppression.

### AdIL-17A transduction in 4T1 cells promotes myelopoiesis in vivo

**Figure 4:**
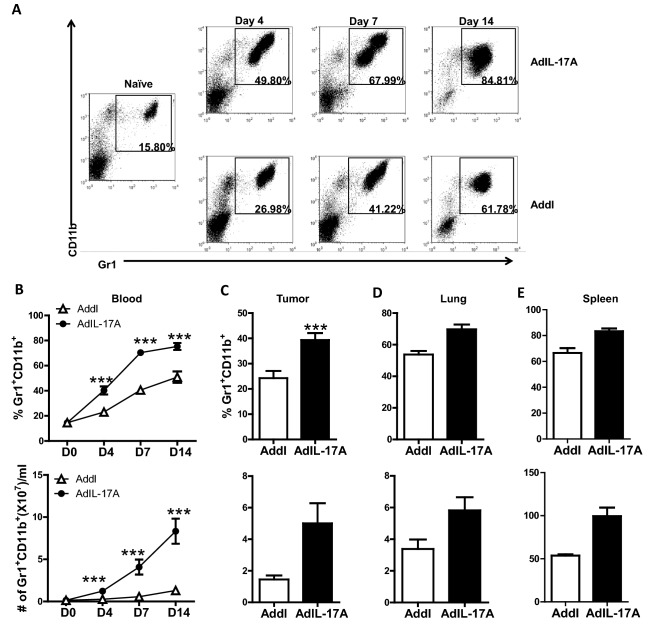
AdIL-17A transduction in 4T1 cells induces rapid expansion of myeloid-lineage immune cells. Mice were treated as described in Fig. 2A. Blood samples, primary tumor, lung, and spleen were collected and immune profiled by flow cytometric analyses (n = 10 per group). (**A**) Representative dot plots showing Gr1^+^CD11b^+^ cells in naïve mice and mice receiving AdIL-17A- or Addl-transduced 4T1 tumors; gated on CD45^+^ cells. (**B**–**E**) Frequency and the absolute number of myeloid Gr1^+^CD11b^+^ cells in peripheral blood, tumor (per gram), lung, and spleen at day 7 post tumor inoculation. *P < 0.05; **P < 0.01; ***P < 0.001 compared to Addl, ^##^*P* < 0.01; ^###^*P* < 0.001 compared to D0, using two-way ANOVA with Dunnett’s multiple comparisons test (panel **B**) or unpaired Student’s *t*-test (panel **C**/**D**/**E**).

Immunosuppressive MDSCs have been shown to accumulate in the 4T1 tumor model^47^. Given that AdIL-17A transduction enhanced the production of cytokines in vitro and/or in vivo that have been implicated in myelopoiesis (G-CSF, GM-CSF, M-CSF, and IL-6)^48^, we sought to characterize the frequency and distribution of MDSCs. The frequency and the absolute number of Gr1^+^CD11b^+^ myeloid cells were significantly increased in tumor-bearing mice compared to naïve mice (Fig. 4). Notably, mice receiving AdIL-17A-transduced 4T1 cells had markedly accelerated kinetics and increased magnitude of Gr1^+^CD11b^+^ cell expansion in peripheral blood compared to control Addl-transduced counterparts. This expansion started as early as four days post tumor inoculation (Fig. 4A,B). Similarly, mice receiving AdIL-17A-transduced 4T1 cells also had elevated frequencies of Gr1^+^CD11b^+^ cells in the primary tumor, lungs, and spleen (Fig. 4C–E). However, the frequencies of Gr1^+^CD11b^+^ cells in the tumor-draining lymph nodes (TDLNs) were extremely low and comparable between the two groups (0.032% vs. 0.034%).

**Figure 5:**
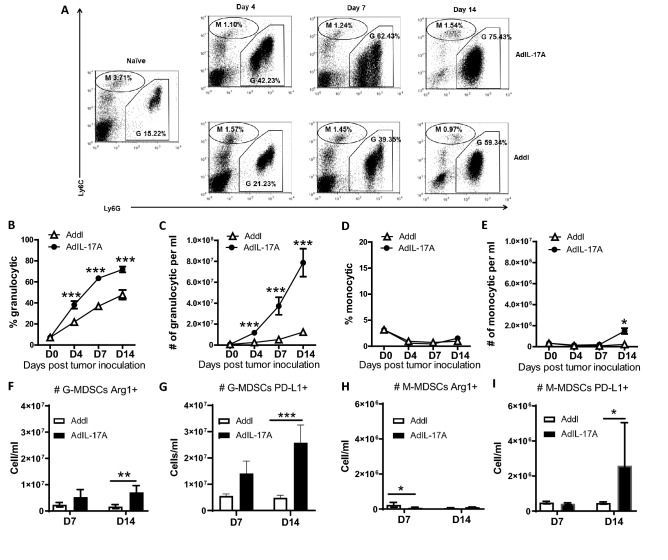
AdIL-17A transduction in 4T1 cells induces expansion of both Ly6G^hi^ and Ly6G^int^ granulocytic populations. Peripheral blood samples were collected from naïve or tumor-bearing mice at days 4, 7, and 14 post tumor inoculation and stained for FACS analysis (n = 10 per group). The analyses were conducted by first gating on CD45^+^ cells. (**A**) Representative dot plots of subpopulations of Gr1^+^/CD11b^+^ cells in naïve, AdIL-17A, and Addl groups. (**B**,**C**) Frequency (out of CD45^+^cells) and the absolute number of granulocytic (G: CD11b^+^Ly6C^int^Ly6G^hi^) cells (**D**,**E**) Frequency and the absolute number of monocytic (M: CD11b^+^Ly6C^hi^Ly6G^lo^) cells. (**F**–**I**) The absolute number of Arg-1- and PD-L1-expressing granulocytic and monocytic GR1^+^/CD11b^+^ cells in AdIL-17A and Addl groups at days 7 and 14 post-tumor inoculation. *P < 0.05; ***P < 0.001 compared to Addl, ^##^*P* < 0.01; ^###^*P* < 0.001 compared to D0, using two-way ANOVA with Dunnett’s multiple comparisons test.

Given that Gr1^+^CD11b^+^ cells represent a heterogeneous myeloid population consisting of granulocytic and monocytic cells^49^, we further analyzed the expression of Ly6G and Ly6C on Gr1^+^CD11b^+^ cells in peripheral blood of naïve and tumor-bearing mice. In naïve mice, granulocytic Ly6C^int^Ly6G^hi^ and monocytic Ly6C^hi^Ly6G^lo^ cells made up approximately 15% and 3.7% of CD45^+^ peripheral blood leukocytes, respectively. Mice bearing AdIL-17A-transduced 4T1 tumors exhibited increased expansion in the frequency and the absolute number of granulocytic cells characterized by low or intermediate expression of Ly6C and intermediate to high expression of Ly6G (Fig. 5A–C). The frequency of monocytic cells, characterized by high Ly6C expression and low Ly6G expression, was very low in all mice; however, the absolute cell number in the AdIL-17A group was significantly higher than the Addl group at day 14 post tumor inoculation (Fig. 5A,D,E). As G-CSF promotes granulocytic myelopoiesis^50^, our results collectively suggest that AdIL-17A transduction in 4T1 tumor cells potently induces expansion of granulocytic myeloid lineage cells. Since MDSCs express multiple immune inhibitory molecules, including arginase 1 (Arg1) and PD-L1^49,51^, we further examined the number of Arg1^+^ and PD-L1^+^ MDSCs in tumor-bearing mice at days 7 and 14 post tumor inoculation. Remarkably, mice receiving AdIL-17A-transduced 4T1 tumors had a massive accumulation of Arg1^+^ and PD-L1^+^ granulocytic and monocytic MDSC populations by day 14 post tumor inoculation (Fig. 5F–I). In total, our results demonstrate that AdIL-17A transduction in 4T1 cells stimulates myelopoiesis and promotes the production of MDSCs in tumor-bearing mice.

### AdIL-17A transduction in 4T1 cells induces potent immune suppression due to quantitative changes in MDSCs

**Figure 6:**
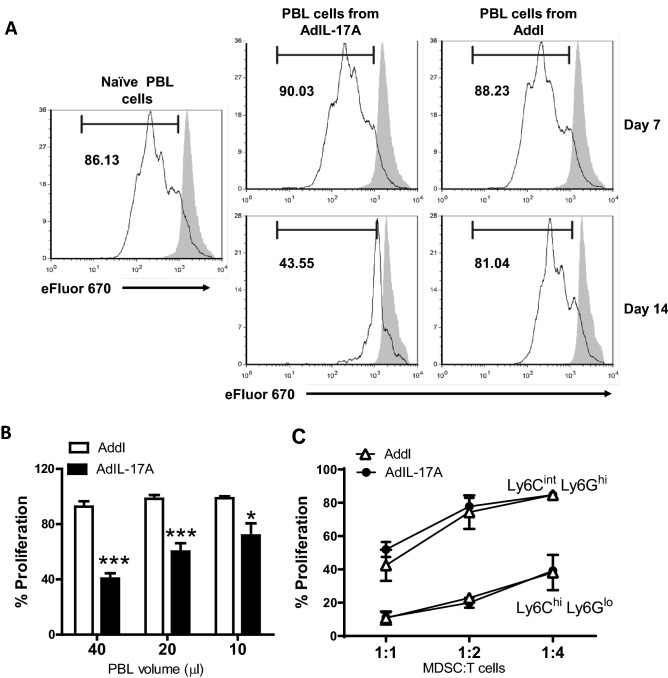
AdIL-17A transduction in 4T1 tumor induces potent immune suppression due to the changes in the quantity of suppressive myeloid cells. Splenic T cells were isolated from the spleen of DO11.10 mouse, labelled with proliferation dye eFluor 670, and co-cultured with bulk peripheral blood leukocytes (PBL) as the course of MDSCs from naïve mice or Ad-transduced tumor-bearing mice or sort-purified myeloid subpopulations. OVA-specific CD4 T cell proliferation in response to OVA peptide stimulation under different culture conditions was measured by flow cytometry based on eFluor 670 dilution. (**A**) Representative histograms of the CD4^+^ T cell proliferation in the presence of PBL recovered from 40 µL of peripheral blood; no peptide stimulation is shown in grey. (**B**) CD4 T cell proliferation in the presence of PBL recovered from 40, 20, and 10 µL of blood collected 14 days after the inoculation of AdIL-17A- or Addl-transduced 4T1 tumor cells (n = 6 per group). (**C**) CD4 T cell proliferation in the presence of graded doses of sort-purified granulocytic cells (CD11b^+^Ly6C^int^Ly6G^hi^) or monocytic cells (CD11b^+^Ly6C^hi^Ly6G^lo^) isolated at day 14 from the spleens of mice inoculated with AdIL-17A- or Addl-transduced tumor cells. *P < 0.05; ***P < 0.001 compared to Addl using unpaired Student’s *t*-test.

Having demonstrated that AdIL-17A-transduced 4T1 tumor cells potently promote the expansion of MDSCs in tumor-bearing mice, we wondered whether tumor-derived IL-17A could alter the quality of MDSCs. To this end, we first compared the suppressive activity of bulk peripheral blood leukocytes isolated from naïve mice or days 7 and 14 after inoculation of AdIL-17A- or Addl-transduced 4T1 tumor cells. Circulating leukocytes isolated from naïve mice and tumor-bearing mice at day 7 post tumor inoculation did not exhibit detectable suppressive activity against the proliferation of naive CD4 T cells (Fig. 6A). In contrast, robust suppressive activity was observed in circulating leukocytes collected at day 14 from mice with AdIL-17A-transduced 4T1 tumors but not Addl-transduced tumors (Fig. 6A,B), implying a potential role of IL-17A in promoting the functional development of MDSCs over time.

Subsequently, the suppressive activities of MDSCs were examined using purified granulocytic (Ly6C^int^Ly6G^hi^) or monocytic (Ly6C^hi^Ly6G^lo^) cells sorted from the spleen of mice 14 days after receiving AdIL-17A-transduced or Addl-transduced 4T1 tumor cells. While both monocytic and granulocytic myeloid cells exhibited dose-dependent suppressive activities against T cell proliferation, the monocytic MDSCs displayed a much higher potency compared to the granulocytic population (Fig. 6C). This observation corresponded to higher frequencies of Arg1-expressing and PD-L1-expressing cells in M-MDSCs compared to their G-MDSCs counterparts (Supplementary Fig. 2). However, the potency of suppression exhibited by monocytic and granulocytic populations was equivalent between the AdIL-17A and Addl groups (Fig. 6C), which also corresponded to comparable levels of Arg-1 and PD-L1 expression on G-MDSCs and M-MDSCs among two treatment groups (Supplementary Fig. 2). Therefore, our data suggest that AdIL-17A-transduction in 4T1 tumors promotes immune suppression by increasing the quantity, but not the suppressive function of MDSCs.

### AdIL-17A-transduced 4T1 tumors elicit distinct organ-specific immune profiles

**Figure 7:**
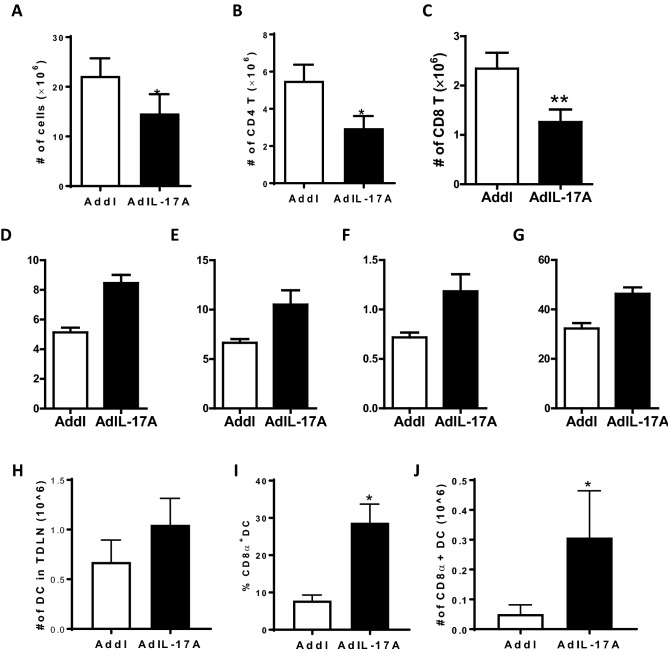
Implantation of AdIL-17A transduced 4T1 cells enhances T cell polarization responses within TDLN. Mice were inoculated with AdIL-17A- or Addl-transduced 4T1 tumor cells on day 0, and TDLNs were collected on day 17. (**A**) The total number of TDLN-derived cells was determined by cell counting. (**B**,**C**) The number of CD4^+^ and CD8^+^ T cells, (**D**–**F**) the frequency of IFN-γ, IL-4 and IL-17A-producing CD4^+^ cells (out of CD4 T cells), and (**G**) the frequency of IFN-γ producing CD8^+^ cells were determined by flow cytometric analyses. (**H**–**J**) The TDLNs were collected, and mononuclear cells were labelled with fluorochrome-conjugated monoclonal antibodies recognizing CD11b, CD11c, Gr1, Ly6c, MHC class-II, CD3, CD4, and CD8α. (**H**) The total number of MHCII^+^CD11c^+^ DCs per TNLD (n = 5 per group); (**I/J**) and the frequency and the absolute number of CD3-CD8α^+^MHCII^+^CD11c^+^ DCs were determined. *P < 0.05; ***P < 0.001 compared to Addl using unpaired Student’s *t-*test.

The observation that AdIL-17A transduction in 4T1 cells induced expansion and accumulation of immune-suppressive MDSCs in multiple organ sites, but not in the TDLNs, prompted us to examine anti-tumor immune responses in the TDLNs. While TDLNs were enlarged in all tumor-bearing mice, we observed a reduced total cell recovery in mice receiving AdIL-17A-transduced 4T1 tumor compared to Addl-transduced counterparts at day 17 post-tumor inoculation (Fig. 7A). The reduced cellularity in TDLNs of the AdIL-17A group was attributed to the loss of CD4 and CD8 T cells, which were significantly diminished compared to the Addl group (Fig. 7B,C). However, intracellular cytokine staining revealed that the frequencies of IFN-γ, IL-4, and IL-17A-producing CD4^+^ T cells, as well as IFN-γ-producing CD8^+^ T cells were significantly higher in the AdIL-17A group compared to the Addl group, indicating that mice receiving AdIL-17A-transduced 4T1 tumors had broad immune activation and differentiation of T cells in the TDLNs (Fig. 7D–G). Notably, an increase in the CD8α^+^ DC population was also observed in the TDLNs of AdIL-17A-transduced tumors compared to the control group at day 4 post tumor inoculation (Fig. 7H–J).

**Figure 8:**
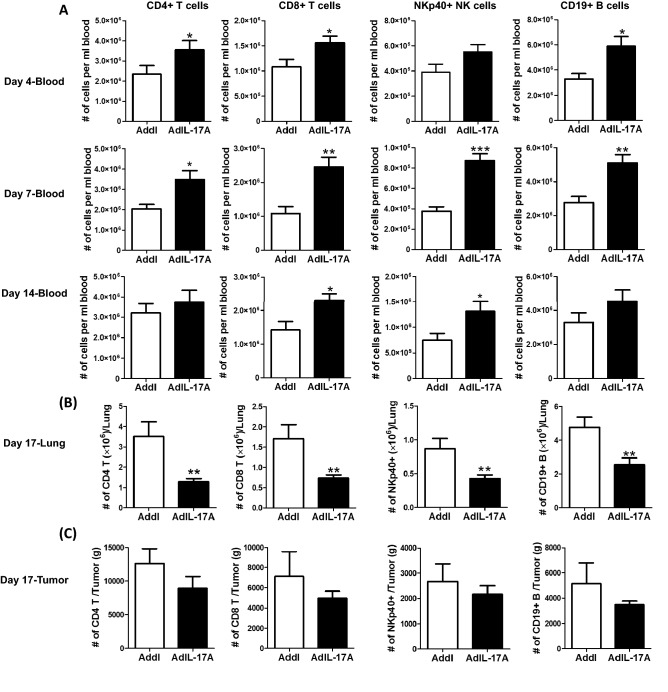
Implantation of AdIL-17A transduced 4T1 tumor cells mobilizes immune cells to the peripheral blood but suppressed infiltration into the lung and tumor sites. Mice were inoculated with AdIL-17A- or Addl-transduced 4T1 tumor cells. Blood samples were collected at days 4, 7, and 14, and mice were sacrificed at day 17 to harvest tissues. The immune profile in blood, lung, and tumor was determined by flow cytometric analyses (n = 10 per group). (**A**) The number of CD4 and CD8 T cells, NK cells, and B cells in peripheral blood samples at days 4, 7, and 14. (**B**,**C**) The number of CD4 and CD8 T cells, NK cells and B cells in the lung and tumor sites at day 17. *P < 0.05; **P < 0.01; ***P < 0.001 compared to Addl using unpaired Student’s *t*-test.

The reduced number of CD4 and CD8 T cells in the TDLNs of the AdIL-17A-transduced 4T1 was likely due to enhanced T cell egress following potent immune activation rather than suppression of immune activation since circulating CD4^+^ and CD8^+^ T cells, as well as NKp40^+^ NK cells and CD19^+^ B cells, were significantly increased in mice receiving AdIL-17A-transduced 4T1 tumor cell compared to the controls (Fig. 8A). Notably, the differences between the two groups were very striking in the peripheral blood at days 4 and 7 post tumor inoculation but became less different by day 14. Remarkably, the immune profiles were completely reversed in the lung (Fig. 8B), with significantly reduced CD4 T cells, CD8 T cells, B cells, and NK cells observed in the AdIL-17A-transduced group compared to the control, suggesting immune suppression in the lung. We also found a trend toward decreased immune cell infiltration into AdIL-17 tumors; however, the differences were not statistically significant. Collectively, our data suggest that AdIL-17A-transduced 4T1 tumor cells elicit immune activation and polarization of immune effector cells in the TDLNs and subsequent egress of effector immune cells. However, the effector functions of immune cells appear to be suppressed at the effector sites, including the lung and tumor, following the expansion of MDSCs.

### Depletion of MDSCs attenuates tumor growth and lung metastasis in mice receiving AdIL-17A-transduced 4T1 tumor cells

**Figure 9:**
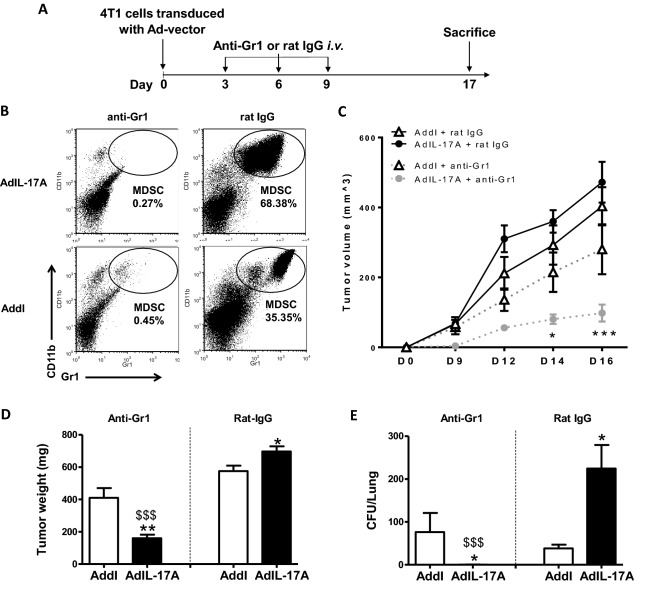
The depletion of MDSCs enhances tumor control in mice receiving AdIL-17A-transduced but not Addl-transduced 4T1 tumor cells. (**A**) Schematic diagram of experimental procedures. Mice were inoculated with 1 × 10^6^ Ad-transduced 4T1 cells at day 0, treated intraperitoneally. with 200 µg of anti-Gr1 or rat-IgG on days 3, 6, and 9, and sacrificed at day 17. (**B**) Representative dot plots demonstrating the impact of anti-Gr1 or rat-IgG on the frequency of Gr1^+^CD11b^+^ cells in peripheral blood at day 7 post tumor inoculation. (**C**) Tumor volumes were measured at days 0, 9, 12, 14, and 16 and depicted for one representative experiment (n = 5 per group). (**D**) Tumor weights and (**E**) lung metastases were determined at day 17. The data are pooled from three individual experiments (n = 10–25 per group). ^###^*P* < 0.001 compared to rat-IgG/AdIL-17A group; *P < 0.05 and **P < 0.01 compared to Addl using two-way ANOVA with Sidak’s multiple comparisons test.

Having shown that AdIL-17A transduction in 4T1 cells induces both immune activation and MDSC-mediated immune suppression, we hypothesized that IL-17A-activated CD4 and CD8 T cells would exert anti-tumor effects if MDSCs were depleted. To this end, we used the Gr1 antibody, which has been widely used to deplete MDSCs in the 4T1 model^52–54^. Anti-Gr1 or rat IgG antibodies were injected intraperitoneally on days 3, 6, and 9 post tumor inoculation (200 µg/dose) (Fig. 9A). On day 7, it was observed that CD11b^+^Gr1^+^ MDSCs in peripheral blood samples were markedly reduced in both groups of mice receiving anti-Gr1, but not rat IgG antibody (Fig. 9B). While depletion of MDSCs markedly attenuated the tumor growth in mice receiving AdIL-17A-transduced 4T1 tumor cells, the anti-Gr1 antibody only displayed a modest inhibitory effect on the growth of Addl-transduced tumors compared to rat IgG-treated counterparts (Fig. 9C,D). Most remarkably, depletion of MDSCs eliminated the lung metastasis of AdIL-17A-transduced 4T1 tumors, but not Addl-transduced tumors (Fig. 9E). Notably, an anti-G-CSF neutralizing antibody also significantly inhibited the MDSC expansion in tumor-bearing mice (Supplementary Fig. 3B,C), highlighting a key functional role of G-CSF in MDSC expansion. In strong agreement with anti-Gr1-mediated MDSC deletion study, anti-G-CSF antibody, but not rat IgG antibody, markedly reduced tumor growth in mice receiving AdIL-17A-transduced, but not Addl-transduced tumors (Supplementary Fig. 3E–G). Together, our results reveal a novel role of tumor-derived IL-17A in potentiating the therapeutic effects of MDSC depletion in attenuating 4T1 tumor growth and metastasis.

## Discussion

IL-17A has complex roles in cancer; hence, understanding how to target IL-17A-enriched tumors is of interest scientifically and practically. In this study, we provide experimental evidence demonstrating an IL-17A production by some human TNBC cell lines and a functional role of tumor-derived IL-17A in concomitantly inducing immune-stimulatory and immune-suppressive responses in a murine model of 4T1 breast cancer. Remarkably, IL-17A expression by tumor cells markedly potentiates the treatment efficacy of anti-Gr1-mediated and anti-G-CSF-mediated MDSC depletion therapies that are otherwise not effective in preventing lung metastasis in 4T1 tumor-bearing mice. Our data strongly support the use of MDSC depletion therapy in treating IL-17A-expressing breast tumor patients.

MDSCs are a heterogeneous population of immature myeloid cells that are prevented from differentiating into macrophages, dendritic cells, and granulocytes^49^. The increased number of MDSCs in tumor-bearing hosts is commonly attributed to aberrant bone marrow myelopoiesis triggered by tumor-derived growth factors such as G-CSF, IL-6, GM-CSF, IL-1β, prostaglandin E2 (PGE2), tumor necrosis factor α (TNFα) and vascular endothelial growth factor (VEGF)^48,55,56^. Previous studies have shown that 4T1 breast mammary carcinoma cells naturally produce G-CSF and GM-CSF, which induce significant expansion of the granulocytic-MDSCs in tumor-bearing hosts^52,57–61^. In our study, many MDSC-inducing molecules, including G-CSF, IL-6, and GM-CSF (Figs. 2, 3), are markedly upregulated in 4T1 culture supernatants upon AdIL-17A transduction comparing to Addl-transduced cells, explaining a rapid expansion of granulocytic-MDSCs in mice receiving IL-17A-expressing 4T1 cells. A similar observation was also made in murine E0771 breast cancer cell line that also induces MDSC production in tumor-bearing C57BL/6 mice^62^. Increased production of G-CSF, GM-CSF, and IL-6 in culture supernatants and enhanced MDSC production in tumor-bearing mice were observed in AdIL-17A-transduced E0771 cells compared to Addl control counterparts (Supplementary Fig. 4). Mechanistically, we showed that increased cytokine/growth factor production and NF-kB activation upon AdIL-17A transduction were attenuated in 4T1-IL-17RCKD cells (Fig. 2), highlighting an autocrine IL-17A-IL-17R-NF-kB pathway in augmenting cytokine/growth factor productions. Additional studies are warranted to investigate whether tumor-derived IL-17A may also stimulate production of these cytokines and growth factors from tumor resident stromal cells and/or tumor resident macrophages through a paracrine pathway. Several molecules, including Arg1, inducible nitric oxide synthase (iNOS), TGF-β, and PD-L1^51,54,57,59^, are shown to mediate the immune suppressive function of MDSCs. In agreement with these understandings, the number of Arg^+^-MDSCs and PD-L1^+^-MDSCs were significantly increased in mice receiving AdIL-17A-transduced tumors in a time-dependent manner (Fig. 5). However, AdIL-17A-transduction in 4T1 tumor cells did not alter the suppressive activity of MDSCs on a per-cell basis (Fig. 6). Therefore, we believe the primary action of tumor-derived IL-17A in tumor-bearing mice is to induce a rapid expansion of myeloid cells, including neutrophils and MDSCs, via the production of STAT3-activating cytokines including IL-6, G-CSF, and GM-CSF by tumor cells. While neutrophils may have both anti- and pro-tumor activities^52,63^, MDSCs, typically, promote tumor angiogenesis, invasion, metastasis, and mediate immunosuppression^49^. Although the bulk myeloid cells expanded in the blood of tumor-bearing mice did not show immunosuppressive activity until 14 days post tumor inoculation, it is increasingly recognized that MDSCs from the tumor microenvironment are more immunosuppressive than those in peripheral blood^64^. As such, immune suppression could likely have occurred before day 14 within the tumor site in mice bearing AdIL-17A-transduced tumors, which had much faster kinetics of MDSC expansion compared to the control group. In breast cancer patients, TNBC samples are found to have a significantly increased number of MDSCs compared to non-TNBC counterparts^65^. Increased expression of the oncogenic p63 isoform has been identified as a culprit for triggering an increased MDSC recruitment through CXCL2- and CCL22-dependent mechanisms^65^. In our study, AdIL-17A transduction also directly upregulated CCR4 expression on tumor cells (Supplementary Fig. 5A) and enhanced expression of two CCR4 ligands, CCL17 and CCL22 in the lung (Supplementary Fig. 5B,C). Therefore, IL-17-producing TNBC cells likely regulate MDSC accumulation in breast cancer patients via a coalition mechanism involving both MDSC production and recruitment.

Tumor-induced MDSCs have been reported to suppress T cell activation and polarization in TDLNs and the tumor microenvironment^64,66^. However, our data suggest that IL-17A-expressing tumors can induce early immune cell activation in the TDLNs, but the immunity is counteracted by robust MDSC-mediated immune suppression in peripheral tissue sites. This notion is supported by the increased frequencies of CD4^+^ effector cells (Th1, Th2, and Th17) and CD8^+^ effector cells in TDLNs (Fig. 7), the unique kinetics of plasma IFNγ levels (Fig. 3), and the impaired accumulation of T cells, B cells and NK cells in the lung and tumor (Fig. 8). While additional experiments are needed to determine the direct contribution of T cells, B cells and NK cells in mediating protective immunity in our study, many previous reports support a role of IL-17A in augmenting T cell responses^21–23^. IL-17A-transfected murine fibrosarcoma cells, but not control tumor cells, have shown to induce tumor-specific CD4 and CD8 T cell responses to protect mice from secondary tumor challenge^21^. In addition, breast cancer patients with high intra-tumoral IL-17A expression have been shown to have strong intra-tumoral Th1 responses^28^. Finally, consistent with a previous study in which IL-17A was shown to increase tumor immunogenicity and immune activation in the TDLNs by promoting DC recruitment^23^, we observed an increased CD8α^+^ DC population in the TDLNs of AdIL-17A-transduced tumors (Fig. 7). Therefore, a possible scenario in our model system is that AdIL-17A-transduction in tumor cells may induce cytokine/chemokine/growth factor production via autocrine and paracrine pathways, leading to a rapid expansion and mobilization of DC and MDSC precursors from bone marrow, which in turn stimulate and inhibit immune responses in a stage and site-specific manner. Since MDSCs were not observed in the TDLNs, AdIL-17A-transduced tumor cells likely elicit immune activation in the TDLNs via an increased DC expansion and antigen presentation at the immune induction phase. However, activated immune effector cells are markedly suppressed or antagonized by the MDSCs in the lung and other sites (Figs. 4, 8). While large numbers of MDSCs in mice with AdIL-17A-transduced tumor cells caused strong immune suppression that resulted in enhanced tumor growth and metastasis, anti-Gr1 antibody and anti-G-CSF therapies were able to effectively control MDSC-mediated tumor growth and lung metastasis (Fig. 9 and Supplementary Fig. 3). In sharp contrast to effective control in AdIL-17A-transduced tumors, both anti-Gr-1 and anti-G-CSF therapies only induced a modest reduction in tumor growth in Addl-transduced tumors and had little effect on lung metastasis (Fig. 9 and Supplementary Fig. 3). A reasonable explaination is that a threshold of cellular immune response is needed for achieving therapeutic effects of MDSC-depletion therapies. Our study collectively demonstrates that MDSC depletion therapy can achieve strong therapeutic effects in IL-17A-rich tumors.

In recent years, MDSC-target therapy has been explored and advanced significantly. In addition to antibody-mediated MDSC depletion, several cytotoxic agents, including doxorubicin, gemcitabine, cisplatin, paclitaxel, and 5-fluorouracil, are also shown to possess functional effects in depleting MDSCs^67–70^. Of interest, TNBC patients with intermediate to high densities of TILs are demonstrated to respond to neoadjuvant chemotherapy treatments^38–40^. In conjunction with the observation that IL-17A^+^ TILs are enriched in TNBC patients^24,25,29^, it is reasonable to speculate that IL-17A-induced anti-tumor immunity is a potential mechanism that works synergistically with neoadjuvant chemotherapy to achieve a favorable clinical response. Recently, multiple breast cancer cell lines are reported to express transcription factor RORγ at mRNA and protein levels, which exhibits a unique role in cholesterol biogenesis in TNBC cells^71^. We queried the Cancer Cell Line Encyclopedia and found approximately 22% of carcinoma cell lines and 54% of breast cancer cell lines express detectable RORγ mRNA (data not shown). Given the critical role of RORγ in mediating IL-17A production by Th17 cells and ILC3^3,4^, it is anticipated that IL-17A can be induced in these RORγ^+^ cells under proper inflammatory conditions. Additional studies are warranted to investigate whether RORγ activators or a low dose of IL-17A can be used for boosting clinical response to neoadjuvant chemotherapy or PD-1 blockade treatment in patients who have a “cold tumor” and normally do not respond to these treatments.

## Materials and methods

### Cell lines and in vitro treatments

Mouse 4T1 and human HS578T mammary carcinoma cells were purchased from ATCC through Cedarlane (Burlington, ON). A stable 4T1 cell line with IL-17RC knockdown (4T1-IL-17RCKD) and its control cell line (4T1-pSMP) was generated and characterized previously^44^. All cell lines were maintained at 37 °C, 5% CO_2_ in Dulbecco's Modified Eagle Medium (DMEM) supplemented with 10% fetal bovine serum (FBS), 100 IU/mL penicillin, 100 µg/mL streptomycin, and 2 mM l-glutamine.

Replication-deficient adenoviral gene transfer vectors expressing murine IL-17A (AdIL-17A), green fluorescent protein (AdGFP), or control virus (Add) were described previously^72,73^. All recombinant adenoviral vectors were amplified, purified, and titrated following established protocols^72^. To determine the dose of adenovirus for transduction, 4T1 cells were seeded in 60 mm cell culture dishes (1 × 10^6^ per dish) overnight and transduced with different doses of AdGFP in PBS containing 1% Ca^2+^ and 1% Mg^2+^ buffer (PBS^+/+^). A multiplicity of infection (MOI) of 200 resulted in approximately 98% transduction efficiency, as determined by GFP expression in tumor cells by flow cytometric analysis. This dose was used for all subsequent transductions. Cells treated with the viral vectors (AdIL-17A or Addl) or PBS had comparable levels of dead cells post-transduction (less than 2%). To determine the level of transgene expression following adenoviral gene transfer, and the impact of transgene expression on tumor growth in vitro, virus-transduced cells were detached and split into 60 mm dishes (5 × 10^5^ per dish) and incubated with 5% CO_2_ at 37 °C for 3 additional days. At 24, 48, and 72 h post-splitting, culture supernatants were collected and stored at − 80 °C for cytokine analysis by ELISA. The cell pellets were stained by PE-conjugated anti-phospho-NFκB p65 (Ser635) antibody (Clone 93H1) (Cell Signalling Technology, Danvers, MA) using intracellular transcription factor staining kit (eBioscience, San Diego, CA). Cell numbers were counted using a hemocytometer. To monitor adenoviral transduction in 4T1 cells, total genomic DNA was isolated 24 h post-transduction using DNeasy Tissue kits (Qiagen, Valencia, CA). Samples were subjected to PCR using primer pairs for Hexon and GAPDH (see Table 1).

**Table 1:** Primers used in the study.

Gene	Forward primer (5′–3′)	Reverse primer (5′–3′)
mG-CSF	GTTGTGTGCCACCTACAAGC	CCATCTGCTGCCAGATGGTGGT
mGM-CSF	ACCACCTATGCGGATTTCAT	TCATTACGCAGGCACAAAAG
mM-CSF	CATCCAGGCAGAGACTGACA	CTTGCTGATCCTCCTTCCAG
mIL-6	GAGGATACCACTCCCAACAGACC	AAGTGCATCATCGTTGTTCATACA
Hexon	AACACCGCCTCCACGCTT	CCAGTGATGGGGTTTCCTTAGTC
GAPDH	CGATGCCCCCATGTTTGTGAT	GCAGGGATGATGTTCTG
hIL-17A	ACCGGAATACCAATACCAATCC	GGATATCTCTCAGGGTCCTCAT
Beta actin	AGC GGG AAA TCG TGC GTG	CAG GGT ACA TGG TGG TGC C

### Mice

Female BALB/c mice were purchased from Charles River Laboratory (Senneville, QC, Canada). Breeding pairs of DO11.10 TCR transgenic mice were purchased from the Jackson Laboratory and bred in-house. All mice were maintained under specific-pathogen-free conditions at the animal facility of the IWK Health Centre and used between 6–12 weeks of age.

### In vivo tumor model and sample collection

Following adenovirus transduction, 4T1 cells treated with PBS, AdIL-17, or Addl were collected at 24 h post-transduction. Cells were washed twice with PBS, resuspended in DMEM, and kept on ice until injection. Tumors were induced in BALB/c mice by inoculating 1 × 10^6^ Ad-transduced 4T1 cells (50 µL volume) into the fourth mammary fat pad. Tumor dimensions were measured using an engineer’s callipers, and the tumor volume was calculated using the equation (½ length × width^2^). Mice were sacrificed on day 17, and the tumor weight and lung metastasis were determined. In some experiments, mice received intraperitoneal injections of anti-Gr1 antibody (clone RB6-8C5) (BioXCell, Lebanon, NH), or rat IgG2b isotype control antibody (200 µg/dose) on days 3, 6, and 9. The depletion of Gr1^+^CD11b^+^ MDSCs was monitored in peripheral blood by flow cytometry 24 h after each intraperitoneal injection.

Blood samples were drawn into heparinized capillary tubes on days 4, 7 and 14 following tumor inoculation. Cells were pelleted by centrifugation, and diluted plasma samples were stored at − 80 °C for cytokine measurements. Red blood cells associated with the cell pellets were lysed in buffer containing 0.15 M NH_4_Cl, 1 mM KHCO_3_, and 0.1 mM EDTA. The remaining white blood cells were washed and resuspended in PBS containing 1% FBS for immune cell phenotyping by flow cytometry. On day 17, after tumor inoculation, mice were sacrificed via isoflurane overdose, and the spleen, TDLN, lung and primary tumor were excised into Hank’s Buffered Salt Solution. Single-cell suspensions were prepared from lymph nodes and spleens as described previously^74,75^. Tumor samples were weighed, mechanically dispersed, and digested with 150 µg/mL collagenase II (Bioshop; Burlington ON, Canada) for 20 min (37 °C, 5% CO_2_). Disaggregated tumor cells were then isolated by filtering through a 70 µm nylon cell strainer (BD Falcon™). The number and phenotype of CD45^+^ tumor-infiltrating leucocytes in each sample were determined by flow cytometric analysis. Similarly, lungs were digested in collagenase IV (0.5 mg/mL) and elastase (5 units/mL) (EPC Elastin Products Company, Owensville, MO) for 75 min at 4 °C, filtered, washed and used for determining immune profile and lung metastasis.

### Colony assay to measure lung metastases

Lung metastasis was quantified by clonal selection assay, as described previously^47^. Briefly, the single-cell lung suspensions were cultured in complete DMEM medium supplemented with 60 µM of 6-thioguanine and seeded into 100 mm tissue culture dishes. Plates were incubated at 37 °C for 10–14 days until colonies were visible. Colonies were fixed with methanol, washed with distilled water, and then stained with 0.03% methylene blue stain. Blue colonies were counted using ImageJ software (NIH, Bethesda, MD). Data are expressed as the total number of metastatic colonies per lung.

### Immune profiling by flow cytometry

To phenotype immune cells in the blood, lymph nodes, spleen, and tumor, approximately 10^6^ cells were first blocked using an anti-mouse CD16/CD32 antibody for 20 min (4 °C) and then labelled with monoclonal antibodies against markers of interest. The antibodies and dyes used in the study include: PE-Texas Red-CD45 (clone 30-F11), PerCPCy5.5-CD3ɛ (clone 145-2C11), FITC-CD4 (clone RM4-5), PE-Cy7-CD8α (clone 53-6.7), eFluor660-NKp40 (clone 29A1.4), PE-CD19 (clone eBio1D3), PE-CD11b (clone M1/70), FITC-Gr1 (clone RB6-8C5), PerCPCy5.5-Ly6c (clone HK1.4), PE-Ly6G (clone 1A8), PE-IFN-γ (clone XMG1.2), PE-IL-17A (clone TC11-18H10), fixable viability dye eFluor™ 450, and PE-IL-4 (clone 11B11), purchased from eBioscience; APC-CD274 (PD-L1) (clone 10F.9G2) was purchased from Biolegend; and FITC-conjugated polyclonal human/mouse Arg1 antibody was purchased from R&D System. Cells were washed and resuspended in fixation buffer (PBS containing 1% formalin). To determine the frequency of Th1, Th2, and Th17 cells, single-cell suspensions were prepared from TDLN and re-stimulated in culture with PMA (20 ng/mL) plus ionomycin (1 µg/mL) (Sigma-Aldrich Co St. Louis, MO) in the presence of Brefeldin A (10 µg/mL) (eBioscience) for 4 h, followed by intracellular cytokine staining as described previously^74,75^. Briefly, the cells were surface labelled first with PercpCy5.5-CD3, FITC-CD4, and PE-Cy7-CD8, then fixed, permeabilized, and intracellularly labeled with PE-IFN-γ, PE-IL-17A, PE-IL-4, or isotype controls. FMO controls were setup accordingly. A total of 100,000 to 500,000 events were collected using a FACSCalibur or FACSAria cytometer (BD Biosciences, Mississauga, ON), and the data were analyzed using FCS Express 6 software (De Novo Software, Los Angeles, CA).

### MDSC suppression assay

To monitor CD4 T cell proliferation and MDSC activity, splenocytes isolated from DO11.10 mice were labeled with the cell proliferation dye eFluor 670 (eBioscience, San Diego, CA)^74^. Labeled cells were resuspended in complete RPMI medium and stimulated with 10 µg/mL OVA_323–339_ peptide (United Biosystems, Bethesda, MA) in a U-bottom 96 well plate (BD Labware, Franklin Lakes, NJ) with or without MDSCs. Plates were incubated at 37 °C for 72 h, and CD4 T cell proliferation was measured via dye dilution by flow cytometry. Splenocytes with and without OVA_323–339_ peptide stimulation were used as positive and negative controls, respectively.

Two different MDSC preparations were analyzed. To evaluate the overall immunosuppressive activity of blood MDSCs, 75 μL of blood was collected from tumor-bearing mice at indicated time points. After red blood cell lysis, the peripheral blood leukocytes were resuspended in 75 µL of RPMI medium. Different volumes of blood leukocytes (40, 20, or 10 µL) were co-cultured with 2 × 10^5^ eFluor 670-labeled splenocytes, as described above. To evaluate the suppressive activity of MDSCs’ subpopulations, granulocytic-MDSCs (CD11b^+^Ly6C^int^Ly6G^hi^), and M-MDSCs (CD11b^+^Ly6C^hi^Ly6G^lo^) were sorted from the spleen of tumor-bearing mice and co-cultured with 2 × 10^5^ of eFluor 670-labeled splenocytes derived from DO11.10 mice at ratios of 1:1, 1:2, and 1:4 (MDSCs:T cells). The proliferation index was calculated by the rate of proliferation of the test sample over the rate of proliferation of positive control.

### ELISA and multiplex Luminex assay

IL-17A and IFNγ levels in plasma samples were determined using the ELISA kit or Luminex assay (eBioscience). IL-17A, G-CSF, GM-CSF, M-CSF, and IL-6 in 4T1 cell culture supernatants were determined using a ProcartaPlex^®^ multiplex Luminex assay (eBioscinece). IFNγ; IL-12p70; IL-13; IL-1β; IL-2; IL-4; IL-5; IL-6; TNFα; GM-CSF; IL-18; IL-10; IL-17A; IL-22; IL-23; IL-27; IL-9; M-CSF and G-CSF were measured in plasma samples collected at day 7 post tumor inoculation using a ProcartaPlex^®^ 19-plex Luminex assay (eBioscience).

### PCR and RT-PCR

Human HS578T TNBC cells were cultured in complete DMEM medium, with or without stimulation of IL-23 (10 ng/mL) (R&D Systems, Minneapolis, MN) and LPS (1 µg/mL) (Sigma Aldrich, Oakville, ON) for various time points. Total RNA samples were isolated using RNeasy Mini Kit (Qiagen, Valencia, CA) and quantified. Approximately 1 µg of total RNA was used for cDNA synthesis using a QuantiTect Reverse Transcription Kit (Qiangen) in a final volume of 20 µL. The IL-17A mRNA level in each sample was determined by quantitative RT-PCR with a volume equivalent to 50–100 ng cDNA using β-actin as an endogenous control. Similarly, the expression of G-CSF, GM-CSF, M-CSF, and IL-6 in Ad-transduced 4T1 cells was also determined by qRT-PCR. Data were collected on RG-6000 Rotor-Gene (Corbett Research, Sydney, Australia). All data were analyzed using 2^−∆∆Ct^ relative quantification technique and expressed relative to GAPDH or β-actin. All of the primer sequences are listed in Table 1.

To monitor adenoviral transduction in 4T1 cells, total genomic DNA was isolated 24 h post-transduction using DNeasy Tissue kits (Qiagen, Valencia, CA). Samples were subjected to PCR using the primer pairs for Hexon and GAPDH.

### Western blotting

Human HS578T TNBC cells were cultured in medium or treated with IL-23 and LPS for various time points. The total protein lysates were prepared using RIPA lysis buffer containing protease inhibitor cocktail (Sigma Aldrich, Oakville, ON), and quantified by Pierce™ BCA Protein Assay Kit (Thermo Fisher Scientific). Approximately 50 µg of total protein was separated by SDS-PAGE. The human IL-17 protein was detected by western blotting, using goat anti-human IL-17A primary antibody (1:500; Ref. PA1-24811, Invitrogen) and anti-goat HRP secondary antibody (1:3,000; sc-2768, Santa Cruz), with the expression level of β-actin (1:100,000; A5384, Sigma) as a loading control.

### Statistical analysis

All data are presented as a mean ± standard error of the mean (SEM) of pooled data from multiple experiments unless indicated otherwise. Data analysis was performed using GraphPad Prism 7 software (GraphPad, La Jolla, CA). The specific statistical analysis is indicated in each figure. *P* values ≤ 0.05 were considered statistically significant.

### Ethical approval

All animal procedures were approved by the Dalhousie University Committee on Laboratory Animals in accordance with the guidelines of the Canadian Council on Animal Care.

## Supplementary information

Supplementary Figures.
