# Supplementary material for: Myeloid-derived suppressor cell depletion therapy targets IL-17A-expressing mammary carcinomas
Source: Sci Rep. 2020 Aug 7;10:13343. doi: 10.1038/s41598-020-70231-7 (PMC7414122; doi:10.1038/s41598-020-70231-7)

Myeloid-derived suppressor cell depletion therapy targets IL-17A-expressing mammary carcinomas

Bassel Dawod[1,2,5], Jinghua Liu[1,2,5,6], Simon Gebremeskel[2,6], , Chi Yan[1,2,6], Antonia Sappong[1,2,5], Brent Johnston[2,3,4,6], David Hoskin[2,3,4,6] Jean Marshall[2,4,6] and

Jun Wang[1,2,3,5,6,*]

[1]Canadian Center for Vaccinology, [2]Department of Microbiology & Immunology, [3]Department of Pediatrics, [4]Department of Pathology, Faculty of Medicine, Dalhousie University, [5]IWK Health Centre, [6]Beatrice Hunter Cancer Research Institute, Halifax, Nova Scotia, Canada

*Correspondence: Dr. Jun Wang, Canadian Center for Vaccinology, IWK Health Centre, Research & Clinical Care Pavilion, 3rd Floor west, 5850/5980 University Avenue, Halifax, NS B3K 6R8, Canada. E-mail: jun.wang@dal.ca, Phone: 1-902-470-7505, Fax: 1-902-470-7590

Running title: Treating IL-17A-positive mammary carcinoma by MDSC depletion

Key words: IL-17A, breast cancer, MDSC, cancer immunotherapy

The authors have no conflicts of interest to declare.

Supplementary Figure 1. Murine mammary carcinomas express transcripts for IL-17RA and IL-17RC. RNA samples were extracted from 4T1 and E0771 tumor cells and reverse transcribed. The GAPDH, IL-17RA and IL-17RC genes were amplified using specific primers as indicated. RNA samples without reverse transcription were used as negative controls for PCR reactions.

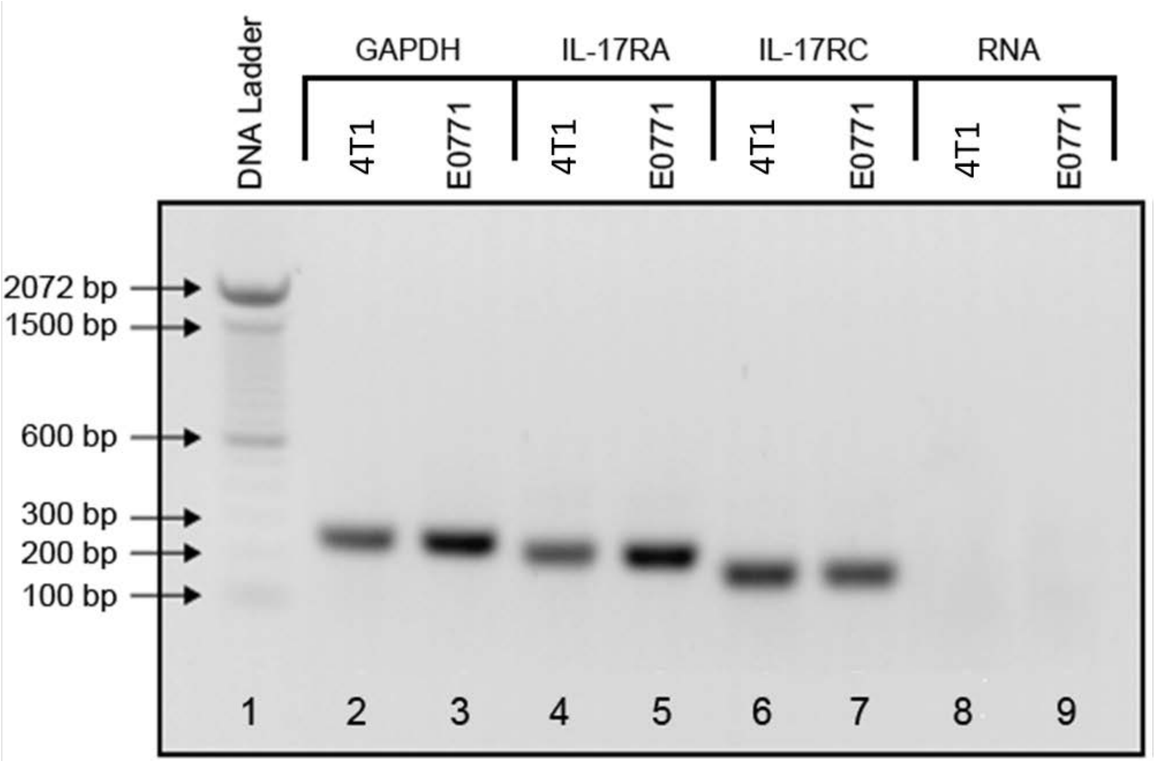

Supplementary Figure 2: Arg1 and PD-L1 are frequently expressed by M-MDSCs in comparison with G-MDSCs. The peripheral blood samples were collected from Addl-transduced or AdIL-17A-transduced tumor-bearing mice at days 7 and 14 post tumor inoculation. The samples were processed and stained with antibodies recognizing CD45, Gr1, CD11b, Ly6C, PDL-1 and Arg1. (A) Representative plots showing the expression levels of Arg1 and PDL-1 on G-MDSC or M-MDSC relative to corresponding isotype controls. (B) The mean fluorescence intensity (MFI) of Arg1 and PDL-1 expression in different subsets of $GR1^{+}CD11b^{+}$ cells following tumor inoculation. The data is presented as the mean ± SEM of 5 mice per group.

A

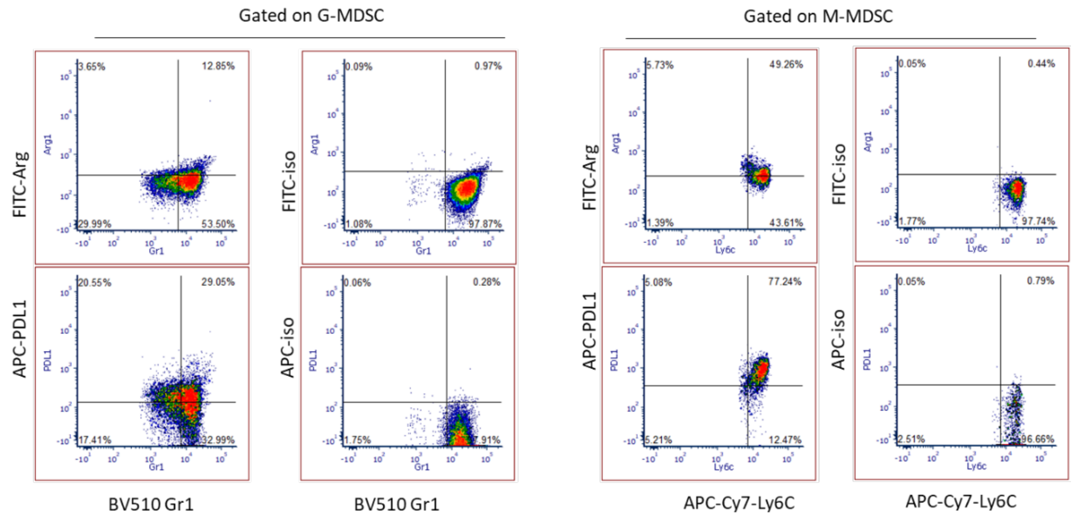

B

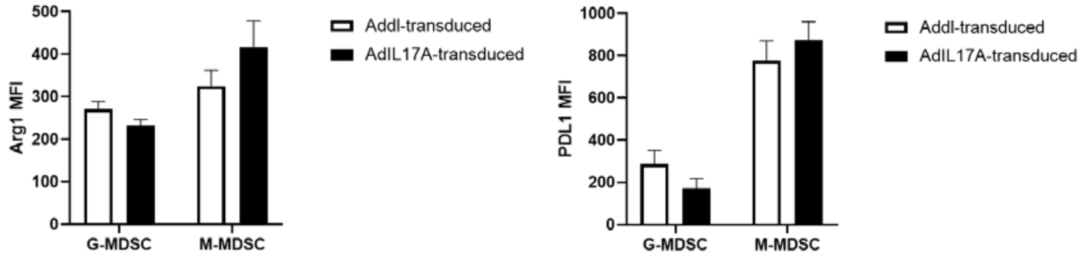

Supplementary Figure 3: **Anti-G-CSF antibody injections markedly reduced MDSC production and tumor growth as well as tumor metastasis in Ad-IL-17A-transduced 4T1 tumor, but not in Addl-transduced tumor. (A)** Schematic diagram of experimental procedures. Mice were inoculated with $1x10^6$ viral-transduced 4T1 cells at day 0, then received three doses (10µg/dose) of anti-G-CSF mAb (R&D system) or rat-IgG at days 3, 6, and 9 and sacrificed at day 17. (**B/C/D**) Absolute numbers of MDSC, G-MDSC, and M-MDSC in peripheral blood samples of different treatment groups. The data is presented as mean ± SEM of 4-10 mice per time point per group. $**P<0.01$ Two-way ANOVA comparison between AdIL-17A/Rat IgG and AdIL-17A/anti-G-CSF groups. (E) Tumor volume was measured at days 0, 5, 7, 9, 11, 13, and 15 (n=5 per group). $*P<0.05$ and $**P<0.01$ Two-way ANOVA comparison between AdIL-17A/Rat IgG and AdIL-17A/anti-G-CSF groups. $\$P<0.05$ and $\$\$P<0.01$ Two-way ANOVA comparison between Addl/Rat IgG and AdIL-17A/anti-G-CSF groups. **(F/G)** Tumor weights and lung metastasis were determined at day 17. $\$ P<0.05$ compared to rat-IgG/AdIL-17A group; $*P<0.05$ and $**P<0.01$ compared to the Addl group using two-way ANOVA using Sidak's multiple comparison test.

A.

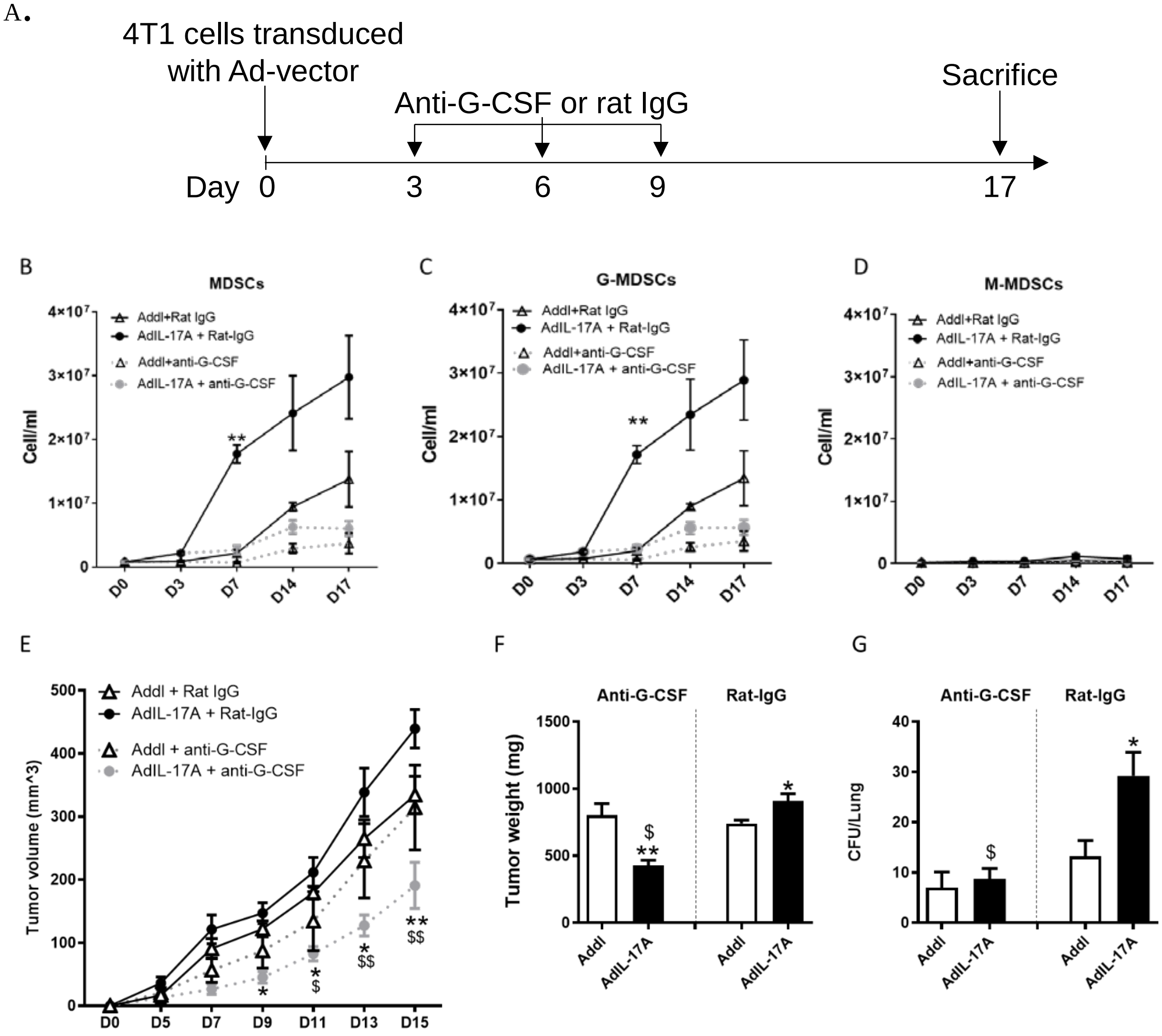

Supplementary Figure 4: **AdIL-17A transduction in E0771 mammary carcinoma results in increased cytokine production *in vitro* and MDSC expansion *in vivo*.** (A) E0771 cells (1×10^6) were transduced with AdIL17A or Addl at MOI=200 in triplicates. The culture supernatants were collected at 24 and 48 hrs post-transduction, and the levels of G-CSF, GM-CSF, and IL-6 were measured by ELISA. (B) Approximately 2 x 10^5 Ad vector-transduced E0771 cells reconstituted in 100 µL Matrigel® (Corning) were injected into the fourth right mammary fat pad of C57BL/6 mice (5 mice per group). Tumor volume was measured at days 4, 7, 12, 14, and 17, and mice were sacrificed at day 17, and the tumor weights were determined. (C/D). Peripheral blood samples were collected at days 0, 4, 14, and 17. The amounts of MDSCs (C) and other immune subsets (D) were determined by flow cytometry. The data are presented as the mean ± SEM of 5 mice. **P*<0.05, ***P*<0.01 and ***p<0.001 compared to the Addl group using two-way ANOVA using Sidak's multiple comparisons tests.

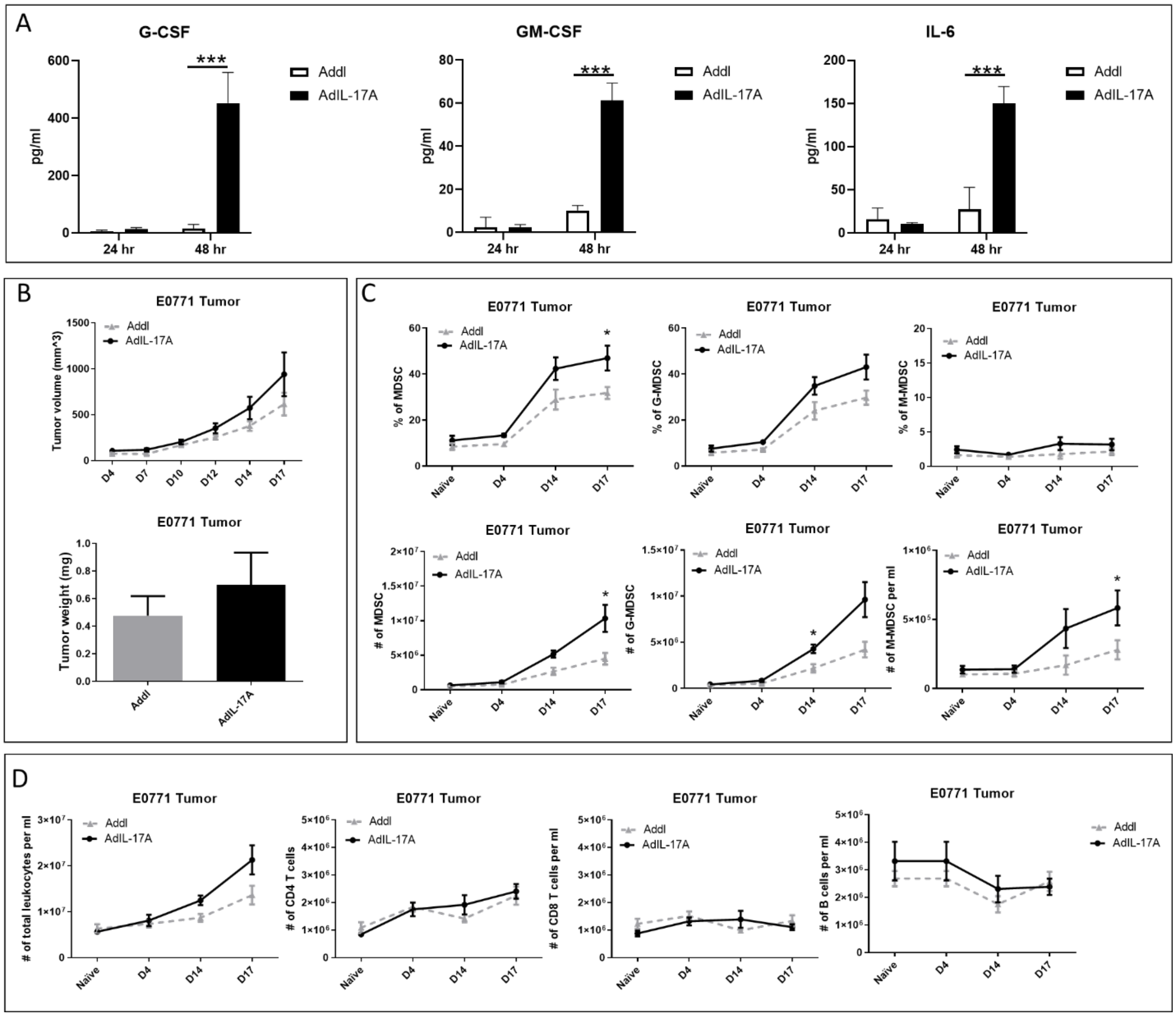

Supplementary Figure 5: **IL-17A transduction in tumor cells induces upregulation of CCR4 and the ligands CCL17/CCL22**. (**A**) 4T1 tumor cells were transduced with Addl or AdIL-17A for 48 hours. The level of CCR4 expression was examined by quantitative RT-PCR. The data was from triplicate samples in each treatment group. (**B/C**) Total RNA was isolated from the lung samples collected from tumor-bearing mice at day 12 post tumor inoculation. The CCL17 and CCL22 levels were examined by quantitative RT-PCR. The data is expressed as the mean ± SEM of 5 mice per group. *P <0.05; **P <0.01 compared to Addl group using unpaired Student's t-test.

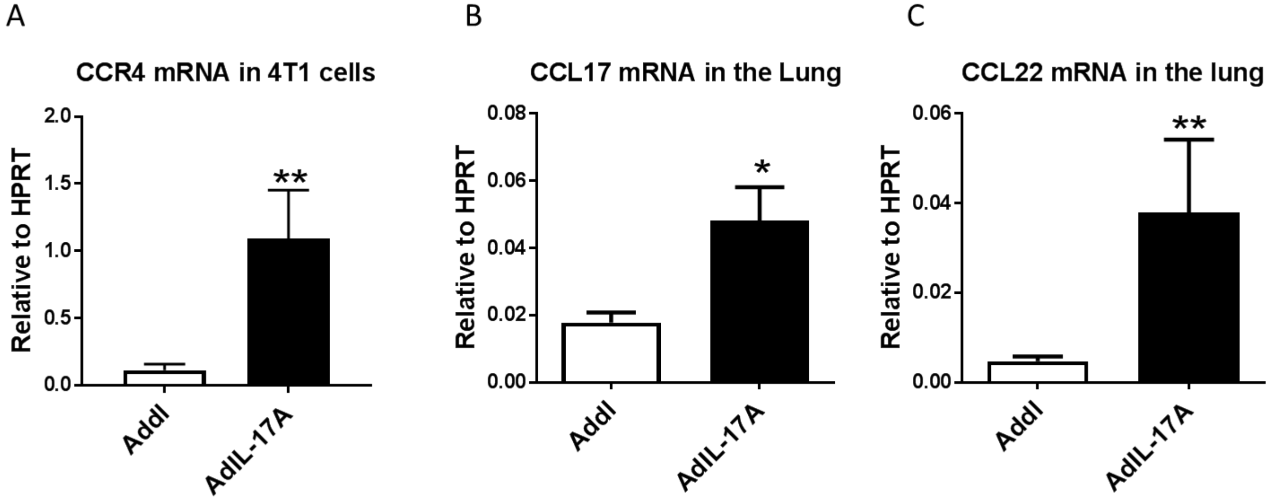

Supplement: Supplementary file 1 — Supplementary Figures. [file 41598_2020_70231_MOESM1_ESM.pdf]
